# State Board of Health Bulletin of Tennessee

**Published:** 1891-01

**Authors:** 


The State Board of Health Bulletin of Tennessee for November 20th

Has a timely article upon the need of a system of Food Inspection by government, and in proof of its argument publishes a private letter written to a grocer in Nashville by a dealer offering to furnish counterfeit coffee grains, to be mixed with genuine coffee, as an adulteration, for a small price per barrel.

There certainly ought to be some means used for checking such dastardly schemes.

